# Supplementary material for: Identification of photoperiod-induced specific miRNAs in the adrenal glands of Sunite sheep (Ovis aries)
Source: Front Vet Sci. 2022 Jul 22;9:888207. doi: 10.3389/fvets.2022.888207 (PMC9354845; doi:10.3389/fvets.2022.888207)
Supplement: Supplementary file 1 [file Data_Sheet_1.ZIP › Supplementary file/miRDeep2 file.zip!/miRDeep2 file/Novel_Structure/SPLP42A2/Novel_293.pdf]

Provisional ID	:	NC_019459.2_5652
Score total	:	2
Score for star read(s)	:	-1.3
Score for read counts	:	0
Score for mfe	:	1.7
Score for randfold	:	1.6
Score for cons. seed	:	
Total read count	:	208
Mature read count	:	206
Loop read count	:	0
Star read count	:	2

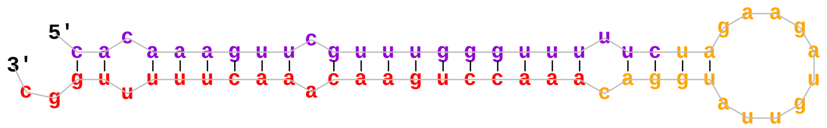

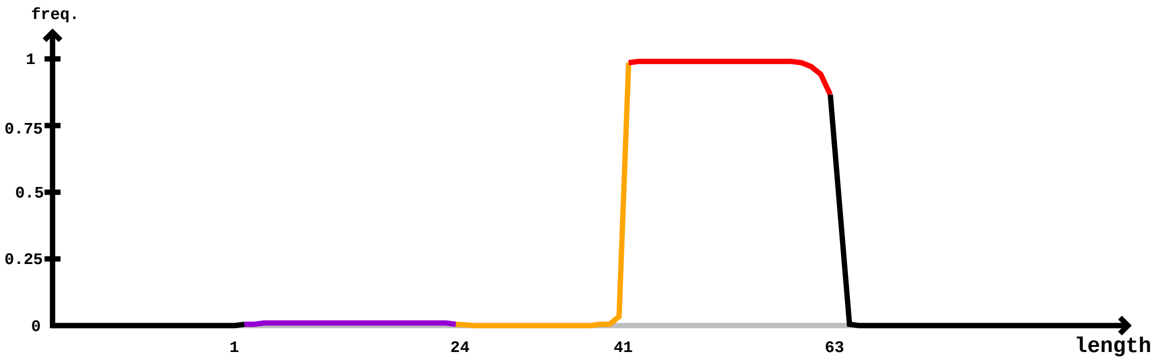

Star **Mature**

```
5'- auaugguauuaaauuggccacaaaguucguuuggguuuuucuagaagauguuauggacaaaccugaacaaacuuuuuggccagcccaauaccauuaggugccaucuacua  -3'   obs
    auaugguauuaaauuggccacaaaguucguuuggguuuuucuagaagauguuauggacaaaccugaacaaacuuuuuggccagcccaauaccauuaggugccaucuacua         exp
    ..((((((((...(((((((.((((((.((((((((((.((((..........)))).)))))))))).)))))).)))))))...))))))))(((((...)))))...   reads   mm   sample
    ..................caAaaaguucguuuggguuuuuc.....................................................................   1       1    seq
    ....................caaaguucguuuggguuuuu......................................................................   1       0    seq
    .......................................................gaAaaaccugaacaaacuuuu..................................   1       1    seq
    .........................................................Aaaaccugaacaaacuuuuu.................................   2       1    seq
    .........................................................Aaaaccugaacaaacuuuuug................................   2       1    seq
    .........................................................Aaaaccugaacaaacuuuuugg...............................   2       1    seq
    ..........................................................aaaUcugaacaaacuuuuu.................................   1       1    seq
    ..........................................................aaaUcugaacaaacuuuuug................................   3       1    seq
    ..........................................................aaaccugaGcaaacuuuuug................................   1       1    seq
    ..........................................................aaaUcugaacaaacuuuuugg...............................   13      1    seq
    ..........................................................aaaccugaaUaaacuuuuugg...............................   1       1    seq
    ..........................................................aaaUcugaacaaacuuuuuggc..............................   177     1    seq
    ..........................................................aaacUugaacaaacuuuuuggc..............................   1       1    seq
    ..........................................................aaaccugaacaaacuuuuuggU..............................   1       1    seq
    ..........................................................Gaaccugaacaaacuuuuuggc..............................   1       1    seq
    ...........................................................aaccugaacGaacuuuuuggcca............................   1       1    seq
```
